# Complete Closure of Both External Auditory Canals by Bone

**Published:** 1890-05

**Authors:** J. W. Carhart

**Affiliations:** Lampasas, Texas


### Original Articles.

☞CONTRIBUTED EXCLUSIVELY TO THIS JOURNAL.☜

*The Articles in this Department are accepted and published with the understanding that we are not responsible for, nor do we indorse the views and opinions of the writers by so doing.*

For Daniel's Texas Medical Journal.

### COMPLETE CLOSURE OF BOTH EXTERNAL AUDITORY CANALS BY BONE.

BY J. W. CARHART, M. D., LAMPASAS, TEXAS.

[Read at Austin District Medical Society.]

IN THE FALL of 1884 I was summoned to treat the wife of Rev. J. G., who was suffering from ear-ache. It was night, and a careful examination of the ear was not made. A few drops of a four per cent. solution of hydrochlorate of cocaine was turned into the ear and a small bag of hop poultice was ordered applied for the balance of the night.

The following day Mr. G. brought his lady to my office, when, on examination, I found the external auditory canal closed. Thinking that adhesion had taken place as the result of inflammatory processes, a silver probe was carefully used, when, to my surprise, I encountered what was evidently true skin covering a bony plate. I then examined the other ear and found that closed in precisely the same manner. I had been acquainted with the lady for some time, but had never heard any complaint of defective hearing, and had never noticed but that her hearing was perfect.

On a test I found that she could hear the watch tick at a distance of five feet. The hearing was not improved by listening with the mouth open, nor was she in the habit of so listening to preaching or in private conversation. She could hear well with her back to the speaker. Her husband had never noticed the peculiarity about her ears; or any defect in her hearing. She had herself never noticed but that her ears were like other people's, and she is satisfied that the condition is congenital. Her family had never spoken to her about it, neither had her family physician, in Illinois, who had treated her from a child. She had never noticed any discharge from the ears, but when she took cold she generally suffered from ear-ache.

I have known the lady, intimately, ever since, and the same condition of things remains with no perceptible change in her hearing powers.

The only theory that, to me, explains her ability to hear, is, that probably the thin bony plate acts as the diaphragm in the mouth-piece of the telephone, which vibrates under the impaction of sound-waves, which vibrations are communicated to the mechanism within, giving the sensation of sound. I have seen but one other similar case reported.

In the Medical Record, for August 3, 1889, Dr. E. E. Holt, of Portland, Me., reports the following case, which is similar, with some points of difference:

"T. M., aged eighteen years, was seen in April, 1889, for an affection of the eye. It was incidentally learned that he had had abscesses in both ears, when seven years old, and the ears discharged more or less for six years, but stopped entirely about five years since. Examinafion showed the canals of both ears of about half the usual length, and occupied by a continuation of the skin of the meatus, with no appearance of membrana tympani. There was complete closure of the canal by what appeared to be bone by all the tests employed.

"The hearing power for the voice was good. The stop-watch was heard only when close to the ear, the tuning fork was heard about ninety seconds, both by bone and ærial conduction. König's rod of thirty thousand vibrations per second was heard by both ears. He heard less distinctly when both ears were closed by pressure on each tragus. Shutting the mouth and closing the nostrils did not seem to affect the hearing power much, if at all. Cases of closure of one meatus with the skin of the canal, continuous over the obstruction, have been observed, but the hearing power is very defective. Cases in which there is a small opening between the exostosis and the walls of the meatus are not uncommon."

